# Nutritional practices and impact of feeding adequacy on clinical outcomes in Chinese respiratory intensive care units patients: a prospective observational study (ORIENT study)

**DOI:** 10.3389/fnut.2025.1719386

**Published:** 2026-01-20

**Authors:** Xu Huang, Yafei Wang, Shuang Geng, Ying Liang, Fan Gao, Tiantian Wang, Huagen Zhang, Jie Fang, Chu Wang, Xiu Gu, Jingping Yang, Zhenshun Cheng, Chun Liu, Wei Lei, Xingang Hu, Hong Luo, Huaihai Fan, Yunhui Zhang, Guoqiang Li, Qingguo Di, Lihua Xing, Yuanyuan Li, Jin Tong, Lin Chen, Wei Sun, Liang Chen, Shengyu Wang, Shaojun Li, Jianhua Liu, Hao Qin, Xiaohua Qiu, Zhuang Ma, Yanxia Li, Hongbin Zhao, Rongzhang Liang, Li Wang, Peng Gao, Linli Sang, Dan Li, Pengguo Hou, Xiuwei Zhang, Dongsheng Wang, Ling Zheng, Feng Hua, Jing Jiang, Xiaoyan Li, Qingrong Nie, Hongwen Zhao, Chuncai Huang, Yingqun Ji, Hong Yu, Liqiang Song, Ju Jin, Ning Li, Weisheng Qian, Jingwu Zeng, Liang Chen, Juan Sun, Wei He, Shuhong Guan, Shukun Chai, Siming Hu, Guanhua Li, Junjie Li, Hongying Jiang, Xuefeng Zhong, Jiashu Li, Jing Zhao, Wei Du, Jiegen Zhang, Zhiliang Liu, Arthur van Zanten, Ye Tian, Ying Cai, Qingyuan Zhan

**Affiliations:** 1 National Center for Respiratory Medicine, State Key Laboratory of Respiratory Health and Multimorbidity, National Clinical Research Center for Respiratory Diseases, Institute of Respiratory Medicine, Chinese Academy of Medical Sciences, Department of Pulmonary and Critical Care Medicine, Center of Respiratory Medicine, China-Japan Friendship Hospital, Beijing, China; 2 Department of Respiratory and Critical Care Medicine, The Central Hospital of Wuhan, Tongji Medical College, Huazhong University of Science and Technology, Wuhan, China; 3 Department of Respiratory and Critical Care Medicine, Peking University Third Hospital, Beijing, China; 4 Medical Research & Biometrics Center National Center for Cardiovascular Diseases Cardiovascular Institute & Fuwai Hospital, The Chinese Academy of Medical Sciences, Beijing, China; 5 Department of Respiratory and Critical Care Medicine, Meizhou People's Hospital, Meizhou, China; 6 Department of Respiratory and Critical Care Medicine, Dezhou People's Hospital, Dezhou, China; 7 Department of Respiratory and Critical Care Medicine, The First Affiliated Hospital of Kunming Medical University, Kunming, China; 8 Department of Respiratory and Critical Care Medicine, The Fourth Affiliated Hospital of China Medical University, Shenyang, China; 9 Department of Respiratory and Critical Care Medicine, Inner Mongolia Baogang Hospital, Baotou, China; 10 Department of Respiratory and Critical Care Medicine, Zhongnan Hospital of Wuhan University, Wuhan, China; 11 Department of Respiratory and Critical Care Medicine, The Third Xiangya Hospital of Central South University, Changsha, China; 12 Department of Respiratory and Critical Care Medicine, The First Affiliated Hospital of Soochow University, Suzhou, China; 13 Department of Respiratory and Critical Care Medicine, Henan Provincial People's Hospital, Zhengzhou, China; 14 Department of Respiratory and Critical Care Medicine, The Second Xiangya Hospital of Central South University, Changsha, China; 15 Respiratory ICU, Tai An City Central Hospital, Tai An, China; 16 Department of Respiratory and Critical Care Medicine, The First People's Hospital of Yunnan Province, Kunming, China; 17 Intensive Care Unit, Medical Center of Chinese People’s Armed Police Force, Tianjin, China; 18 Department of Respiratory and Critical Care Medicine, Cangzhou Central Hospital, Cangzhou, China; 19 Department of Respiratory and Critical Care Medicine, The First Affiliated Hospital of Zhengzhou University, Zhengzhou, China; 20 Department of Respiratory and Critical Care Medicine, Xiangya Hospital, Central South University, Changsha, China; 21 Department of Respiratory and Critical Care Medicine, The Second Affiliated Hospital of Chongqing Medical University, Chongqing, China; 22 Department of Respiratory and Critical Care Medicine, Sichuan Provincial People’s Hospital, Chengdu, China; 23 Department of Respiratory and Critical Care Medicine, No.2 People's Hospital of Fuyang City, Fuyang, China; 24 Department of Respiratory and Critical Care Medicine, The Affiliated Huai'an No.1 People's Hospital of Nanjing Medical University, Nanjing, China; 25 Department of Respiratory and Critical Care Medicine, The First Affiliated Hospital of Xi'an Medical University, Xi'an, China; 26 Department of Respiratory and Critical Care Medicine, The First Affiliated Hospital of Xi'an Jiaotong University, Xi'an, China; 27 Department of Respiratory and Critical Care Medicine, The First Affiliated Hospital of Hebei North University, Shijiazhuang, China; 28 Department of Respiratory and Critical Care Medicine, The First Affiliated Hospital of Second Military Medical University, Shanghai, China; 29 Department of Respiratory and Critical Care Medicine, The Affiliated Drum Tower Hospital of Nanjing University Medical School, Nanjing, China; 30 Department of Respiratory and Critical Care Medicine, The General Hospital of Northern Theater Command, Shenyang, China; 31 Department of Respiratory and Critical Care Medicine, The First Affiliated Hospital of Dalian Medical University, Dalian, China; 32 Department of Respiratory and Critical Care Medicine, Dali Bai Autonomous Prefecture People's Hospital, Dali, China; 33 Department of Respiratory and Critical Care Medicine, Longyan First Affiliated Hospital of Fujian Medical University, Longyan, China; 34 Department of Respiratory and Critical Care Medicine, Nanjing First Hospital, Nanjing Medical University, Nanjing, China; 35 Department of Respiratory and Critical Care Medicine, The Second Hospital of Jilin University, Changchun, China; 36 Department of Respiratory and Critical Care Medicine, Northern Jiangsu People's Hospital, Yangzhou, China; 37 Department of Respiratory and Critical Care Medicine, The First Hospital of Jilin University, Changchun, China; 38 Department of Respiratory and Critical Care Medicine, The Third People's Hospital of Datong, Datong, China; 39 Department of Respiratory and Critical Care Medicine, The Affiliated Jiangning Hospital of Nanjing Medical University, Nanjing, China; 40 Department of Respiratory and Critical Care Medicine, The First Affiliated Hospital of USTC (University of Science and Technology of China), Hefei, China; 41 Department of Respiratory and Critical Care Medicine, Second Affiliated Hospital of Anhui Medical University, Hefei, China; 42 Department of Respiratory and Critical Care Medicine, Huzhou Central Hospital, Huzhou, China; 43 Department of Respiratory and Critical Care Medicine, The Affiliated Yantai Yuhuangding Hospital of Qingdao University, Yantai, China; 44 Department of Respiratory and Critical Care Medicine, Shanxi Bethune Hospital, Taiyuan, China; 45 Department of Respiratory and Critical Care Medicine, Beijing Fangshan Liangxiang Hospital, Beijing, China; 46 Department of Respiratory and Critical Care Medicine, The First Hospital of China Medical University, Shenyang, China; 47 Intensive Care Unit, The Second Hospital of Tianjin Medical University, Tianjin, China; 48 Department of Respiratory and Critical Care Medicine, East Hospital Affiliated To Tongji University, Shanghai, China; 49 Department of Respiratory and Critical Care Medicine, Guizhou Provincial People's Hospital, Guiyang, China; 50 Department of Respiratory and Critical Care Medicine, The First Affiliated Hospital of Air Force Military Medical University, Xi’an, China; 51 Department of Respiratory and Critical Care Medicine, The Second People's Hospital of Wuhu, Wuhu, China; 52 Department of Respiratory and Critical Care Medicine, The Fourth Affiliated Hospital Zhejiang University School of Medicine, Yiwu, China; 53 Department of Respiratory and Critical Care Medicine, The Second Affiliated Hospital of AirForce Military Medical University, Xi’an, China; 54 Department of Respiratory and Critical Care Medicine, Jingzhou Hospital Affiliated to Yangtze University, Jingzhou, China; 55 Department of Respiratory and Critical Care Medicine, Beijing Jingmei Group General Hospital, Beijing, China; 56 Department of Respiratory and Critical Care Medicine, The First Affiliated Hospital of Anhui Medical University, Hefei, China; 57 Department of Respiratory and Critical Care Medicine, The Central Hospital Affiliated to Shenyang Medical College, Shenyang, China; 58 Department of Respiratory and Critical Care Medicine, The First People’s Hospital of Changzhou, Changzhou, China; 59 Department of Respiratory and Critical Care Medicine, Shijiazhuang People's Hospital, Shijiazhuang, China; 60 Department of Respiratory and Critical Care Medicine, Suzhou Municipal Hospital, Suzhou, China; 61 Department of Respiratory and Critical Care Medicine, Tianjin Chest Hospital, Tianjin, China; 62 Department of Respiratory and Critical Care Medicine, Ruijin Hospital, Shanghai Jiaotong University School of Medicine, Shanghai, China; 63 Department of Respiratory and Critical Care Medicine, Beijing Rehabilitation Hospital of Capital Medical University, Beijing, China; 64 Department of Respiratory and Critical Care Medicine, Beijing Hospital, Beijing, China; 65 Department of Respiratory and Critical Care Medicine, The First Hospital of Lianyungang, Lianyungang, China; 66 Department of Respiratory and Critical Care Medicine, The Second Hospital of Hebei Medical University, Shijiazhuang, China; 67 Department of Respiratory and Critical Care Medicine, General Hospital of Southern Theater Command, PLA, Guangzhou, China; 68 Department of Respiratory and Critical Care Medicine, Zhuozhou City Hospital, Zhuozhou, China; 69 Department of Respiratory and Critical Care Medicine, Tangshan Center Hospital, Tangshan, China; 70 Department of Intensive Care Medicine & Research, Gelderse Vallei Hospital, Ede, Netherlands; 71 Division of Human Nutrition and Health, Chair Group Nutritional Biology, Wageningen University & Research, Wageningen, Netherlands

**Keywords:** energy intake, enteral nutrition, feeding adequacy, mortality, nutritional status, RICU

## Abstract

**Background:**

The current status of nutritional support for patients in Respiratory ICU across mainland China remains inadequately characterized. This multi-center study, conducted in RICUs, was designed to investigate nutritional practices in this specific patient population, focusing on initiation timing, energy/protein adequacy, and associations with clinical outcomes.

**Methods:**

A prospective, observational study enrolled 1,026 patients (ICU stay >48 h) across 68 Chinese RICUs. We analyzed EN initiation rates (24 h/48 h), caloric/protein intake adequacy during the first 7 days, and outcomes via multivariable Cox regression.

**Results:**

EN initiation occurred in 36.8% (24 h) and 43.4% (48 h) of patients. Excluding patients who could take oral food, the proportion of patients who started EN within 24 and 48 h increased to 86.9 and 95.3%. Among 499 EN-fed patients, 26.1% developed EN complications. Caloric analysis (*n* = 317) identified three trajectories: underfeeding (<70% targets, 32.5%), adequate feeding (70–110, 43.2%), and overfeeding (>110, 24.3%). Overfeeding independently predicted higher non-social infections and significantly increased mortality risk in patients >50 years (HR = 1.83, 95% CI 1.02–3.28; *p* = 0.04). Mean protein intake was 0.9 g/kg/day, with no 28-day mortality benefit at higher thresholds (≥1.2 g/kg: *p* = 0.31; ≥1.3 g/kg: *p* = 0.42).

**Conclusion:**

This multicenter study demonstrates optimal early EN initiation rates in Chinese RICUs. Energy overfeeding was associated with increased mortality risk only in patients >50 years and non-social infections risk, whereas protein adequacy showed no outcome associations. Protocolized EN delivery balancing adequacy and overfeeding risks is urgently needed in RICUs.

**Clinical trial registration:**

Identifier, NCT04958447.

## Background

Critically ill patients admitted to intensive care units (ICUs) frequently encounter nutritional challenges stemming from the hypercatabolic state induced by acute critical illness. During this acute phase, the body initiates tissue catabolism to generate metabolic substrates that support essential acute-phase physiological priorities, including hemostatic regulation and immune defense mechanisms.

This hypercatabolic state, however, may precipitate significant protein-energy deficits in this population, which has been strongly correlated with detrimental clinical outcomes and substantial healthcare expenditures. This cascade manifests clinically as heightened susceptibility to non-social infections, prolonged ventilator dependence, extended ICU hospitalization, and elevated mortality risk (1–3).

Despite the pervasive issue of malnutrition in ICU settings, this metabolic derangement frequently remains underrecognized and persists as a global health challenge. Nutrition, especially enteral nutrition (EN), has been widely accepted as the standard care for critically ill patients (4–7). EN is considered more physiologically appropriate, helps maintain gastrointestinal integrity, is easy to administer, and is cost-effective (8, 9). Growing clinical evidence shows that targeted nutritional support can help patients in several ways. It maintains gut health, repairs tissue, regulates the immune system, reduces excessive metabolism after injury, and lowers infection risk. Together, these benefits help patients recover faster and shorten hospital stays (10).

The volume of scholarly publications in clinical nutrition has expanded considerably in recent years. Nevertheless, current evidence remains marked by persistent controversies and unresolved questions regarding critical care feeding protocols. Fundamental questions remain regarding the use of early enteral versus parenteral nutrition in high-risk patients, optimal feeding timing for unstable patients, and the value of gastric residual volume monitoring. Furthermore, there is a significant gap between guideline recommendations and clinical practice, as evidenced by inadequate enteral nutritional intake (11) and unnecessary feeding interruptions (12–14). While RICU patients demonstrate overlapping clinical profiles with general ICU populations, they present distinct pathophysiological characteristics that necessitate prioritized management of oxygenation deficits, carbon dioxide retention disorders, acid–base homeostasis disturbances, and severe pulmonary infections. These compounding barriers pose significant challenges to delivering effective nutritional interventions and achieving prescribed caloric and protein requirements. There is a lack of epidemiological data, specifically from RICUs, particularly in China.

This multicenter prospective observational study constitutes the largest systematic investigation to date specifically targeting nutritional support practices in RICUs across China. The primary objective is to establish comprehensive epidemiological data regarding EN administration protocols in Chinese RICU settings. A secondary analysis of this dataset specifically examined the relationship between nutritional adequacy levels during the first week following ICU admission and key clinical outcomes in critically ill respiratory patients.

## Materials and methods

### Study design

This prospective observational investigation was implemented across 68 RICUs spanning multiple provinces in mainland China from July 1 (00:00) to July 21 (24:00), 2021. Demographic and clinical parameters were systematically documented at RICU admission, with patient outcomes evaluated at 28 days post-enrolment. Data acquisition utilized standardized electronic case report forms (eCRFs) administered through a secure web-based platform.

The study was approved by the ethics committee of China-Japan Friendship Hospital (approval no. 2021–38-K22), and written informed consent was obtained from all patients or their legal representatives. The inclusion criteria were as follows:

1. Patients admitted to the RICU who survived for more than 48 h.
2. Patients aged 18 years or older.

The exclusion criteria were as follows:

1. Severe trauma cases (defined as life-threatening trauma requiring emergency surgery and treatment, including criteria such as low systolic blood pressure, abnormal respiratory rate or heart rate, serious disturbance of consciousness, traumatic limb amputation, flail chest, multiple long bone fractures, or fall injuries from a height of more than 3 meters).
2. Surgical patients admitted to the RICU.
3. Patients or their legal representatives who declined to participate in the study.

### Data collection

Standard demographic variables including age, sex, admission source, weight, and height were systematically documented. Within 24 h of RICU admission, Acute Physiologic and Chronic Health Evaluation II (APACHE II) scores and Sequential Organ Failure Assessment (SOFA) scores were calculated.

### Nutritional risk status

Nutritional risk stratification was performed using the modified Nutrition Risk in Critically Ill (mNUTRIC) score and Nutritional Risk Screening 2002 (NRS 2002) instruments, with scores ≥5 defining the high-risk cohort according to standardized clinical thresholds. Routine laboratory tests were conducted to measure plasma indicators like albumin and proalbumin.

### Feeding performance

The primary objective was to evaluate both the timing of nutritional support initiation (within 24 and 48 h) and the modalities employed, including oral intake versus medical nutrition therapy encompassing EN, total parenteral nutrition (TPN), or supplemental parenteral nutrition (SPN). Energy and protein requirements for each nutritional intervention were systematically recorded. EN was defined as feeding through tubes, excluding oral feeding, which was measured separately. PN is any peripheral or central intravenous administration of nutrition. Details of medical nutrition delivery were recorded within the first 7 days of RICU admission. Enteral feeding volume, energy density of EN formula, feeding routes, and feeding styles were documented. PN volume and composition of nutrients (carbohydrates, proteins, and fats) were also recorded.

### Feeding tolerance and complications

Hemodynamic and gastrointestinal function parameters were evaluated before EN initiation. Measures to reduce the risk of aspiration during EN administration, such as bed head angle, GRV, use of gastrointestinal motility drugs, tracheal tube balloon pressure in patients with artificial airways, and nasojejunal tube usage, were recorded. Reasons for discontinuing EN within the first 7 days of RICU admission were documented. Intolerance to EN included symptoms like nausea, vomiting, aspiration, abdominal pain and distension, high GRV, and diarrhea.

### Feeding adequacy

Current critical care protocols advocate early nutritional intervention initiation (typically within 24–48 h post-admission) for hemodynamically stabilized patients. Nevertheless, during the initial 48-h window following RICU admission, suboptimal nutrient delivery frequently occurs secondary to compromised hemodynamic profiles and disease severity. By the third post-admission day, when physiological parameters demonstrate progressive stabilization, both caloric and protein provisions achieve therapeutic consistency. Consequently, we designated the mean energy/protein delivery during days 3–5 as established clinical parameters for nutritional assessment.

Energy requirements were calculated based on body weight and adjusted for BMI to better reflect metabolic needs. The following calculations were applied: BMI 25–30 kg/m^2^: 25–30 kcal/kg/day; BMI 30–50 kg/m^2^: 11–14 kcal/kg/day; BMI > 50 kg/m^2^: 22–25 kcal/kg/day. Adequate feeding was defined as achieving 70–110% of the required energy intake. Energy underfeeding refers to intake below 70%, and overfeeding indicates intake above 110% (7). The recommended protein intake ranges from 1.2 to 2.0 g/kg/day (4).

### Clinical outcomes

Clinical outcomes, recorded at 28 days after RICU admission, included survival status (alive, dead, or lost), non-social infections, days free from mechanical ventilation, and RICU-free days.

The dataset contained missing values, and single imputation (EM) was performed to address these missing values.

### Statistical analysis

Descriptive analyses were conducted using appropriate statistical measures: categorical variables were expressed as frequency distributions (%) while continuous variables were reported as mean ± standard deviation or median with interquartile range (IQR). Missing data were handled without assumptions, and the characteristics of patients with missing outcomes were compared to those with complete data.

Proportions were compared using χ^2^ or Fisher’s exact tests for categorical variables, while the t-test or Wilcoxon rank sum test was used for continuous variables, as appropriate. Multivariable Cox proportional hazards analyses were performed to evaluate the association between feeding adequacy and 28-day mortality, while logistic regression models were employed to assess relationships with other clinical outcomes (e.g., hospital-acquired infections, ICU length of stay). A two-sided *p*-value of 0.05 or less was considered statistically significant. Unless otherwise specified, statistical analyses were performed using SPSS software, version SPSS 26.

## Results

### ICU and hospital characteristics

The multicenter investigation encompassed 68 RICUs across general hospitals (see Supplementary Table 1). Notably, only 13.2% of participating RICUs employed professional dietitians, while 32.4% maintained established multidisciplinary nutritional support teams.

### Characteristics and outcomes of enrolled patients

**Figure 1:**
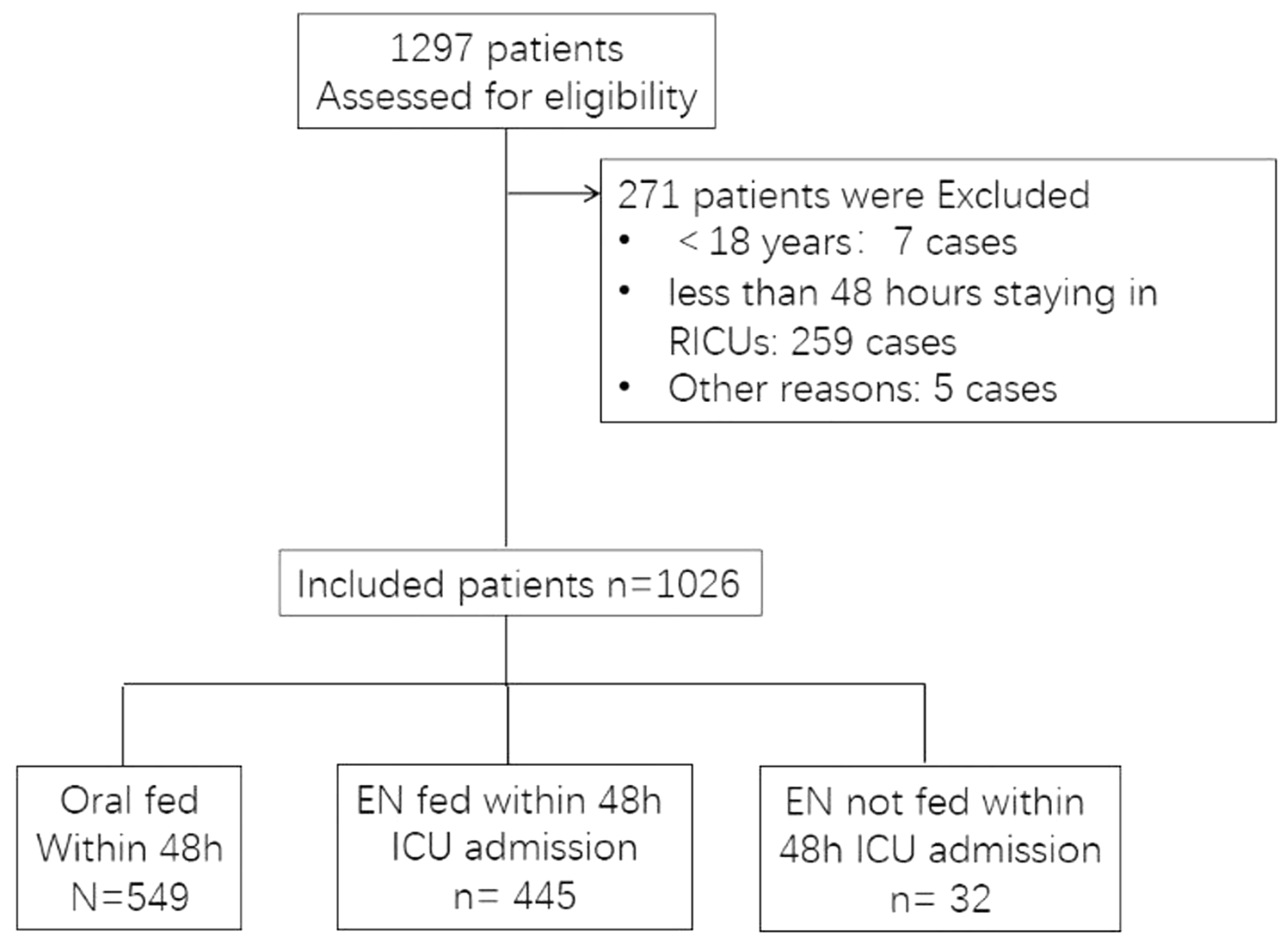
Flow of patients recruitment.

From an initial screening cohort of 1,297 patients, 1,026 met inclusion criteria (Figure 1). Table 1 summarizes baseline characteristics and clinical parameters of the study cohort. Among them, the mean age was 70.0 ± 14.1 years. The median APACHE II score and SOFA scores were 14 (10.19) and 4 (2.6), respectively. The median ventilator-free days (VFDs) and ICU-free days at 28 days were 28 days (20.28) and 17 days (3.22), respectively. The 28-day mortality rate was 13.7% (141/1026), and non-social infections occurred in 7.2% (74/1026) of patients.

**Table 1:** Baseline of patient characteristics.

Variable	*n* = 1,026(%)
Age, mean(SD), y	70 ± 14.1
Male	690 (67.3)
BMI, mean (SD), kg/m^2^	22.34 ± 4.19
BMI<18.5	170 (16.6)
18.5 ≤ BMI <30	812 (79.1)
BMI ≥ 30	44 (4.3)
APACHEII score, median (Q1, Q3)	14 (10.19)
SOFA score, median (Q1, Q3)	4 (2.6)
GCS score, median (Q1, Q3)	15 (11.15)
Simplified NUTRIC score	3 (2.4)
Simplified NUTRIC score ≥5	256 (25.0)
NRS-2002 score	6 (4.7)
NRS-2002 score (≥5)	686 (66.9)
NRS ≥ 5 and NUTRIC ≥5	213 (20.8)
NRS ≥ 5 or NUTRIC ≥5	729 (71.1)
Causes of ICU admission	
Severe penumonia	407 (39.7)
Extrapulmonary sepsis	27 (2.6)
COPD	241 (23.5)
ARDS	18 (1.8)
Malignant tumor	86 (8.4)
Albumin	32.6 ± 5.8
Proalbumin*	142.9 ± 77.1
Patients not measured Proalbumin (%)	407 (39.7)
VFDs	28 (20.28)
ICU-free days	17 (3.22)
Number of deaths	141 (13.7)
Non-social infections	74 (7.2)

Data are expressed as mean (SD), or n (%). BMI, body weight index; APACHE, acute physiology and chronic health evaluation; SOFA, sequential organ failure assessment, GCS, Glasgow coma score; COPD, chronic obstructive pulmonary disease; ARDS, acute respiratory distress syndrome; VFDs, ventilator-free days. *Proalbumin values were missed in 39.8% patients.

### Nutrition risk score

The median mNUTRIC and NRS2002 scores were 3 (2.4) and 6 (4.7), respectively. Based on these assessments, 256 patients (25.0%) and 686 patients (66.9%) met criteria for high nutritional risk via the mNUTRIC and NRS2002 scales, respectively. Within the cohort of 1,026 critically ill patients, 170 (16.6%) exhibited malnutrition upon ICU admission, operationally defined as a body mass index (BMI) < 18.5 kg/m^2^. Biochemical analysis revealed mean serum albumin and prealbumin concentrations of 32.6 ± 5.8 g/L and 142.9 ± 77.1 g/L, respectively (Table 1).

### Nutritional support

Within the initial 7 days following RICU admission, nutritional support was initiated in 1,018 patients (99.2% of the cohort). Of these, 97.8% received oral intake and/or EN, with SPN administered in combined regimens. Timely nutritional intervention (≤48 h post-admission) was implemented in 88.6% of cases (909/1026). The modalities of nutritional support during the critical 48-h window demonstrated the following distribution: exclusive oral feeding (37.5%), EN monotherapy (35.2%), PN alone (4.8%), combined EN + PN (9.1%), oral+EN coadministration (2.3%), oral+PN supplementation (6.7%), and oral intake with oral nutritional supplements (4.3%).

### EN initiation

During the initial 7-day period following RICU admission, 499 patients received EN, with 378 (75.8%) and 445 (89.2%) initiating EN therapy within 24 and 48 h of RICU admission, respectively. Among patients stratified as high nutritional risk by mNUTRIC scoring, EN initiation occurred within 24 h in 138 cases (53.9%) and within 48 h in 154 cases (60.2%). Conversely, for those categorized as high nutritional risk through NRS2002 assessment, timely EN initiation was achieved in 306 cases (44.6%) within 24 h and 337 cases (49.1%) within 48 h.

Shock was observed in 12.8% of the cohort upon EN initiation. Among patients who commenced EN within the initial 7-day period, 67.6% had intact gastrointestinal (GI) function, 23.2% were in acute gastrointestinal injury grade (AGI) I, 7.9% were in AGI II, 1.2% were in AGI III, and none were in AGI IV at the time of EN initiation. The most common reasons for not initiating EN within 48 h were oral feeding, uncontrolled shock, and uncontrolled upper gastrointestinal bleeding (Table 2).

**Table 2:** The characteristics of initiating EN in patients in the RICU.

Index	Results (*n* = 1,026)
EN initiation within 7d	499 (48.6%)
EN initiation within 24 h of RICU admission	378 (36.8%)
EN initiation within 48 h RICU admission	445 (43.4%)
EN initiation within 24 h of RICU admission in high nutritional risk patients (mNUTRIC ≥5; 256)	138 (53.9%)
EN initiation within 48 h of RICU admission in high nutritional risk patients (mNUTRIC ≥5; 256)	154 (60.2%)
EN initiation within 24 h of RICU admission in high nutritional risk patients (NRS2002 ≥ 5; 686)	306 (44.6%)
EN initiation within 24 h of RICU admission in high nutritional risk patients (NRS2002 ≥ 5; 686)	337 (49.1%)
AGI on EN initiation	491
Gastrointestinal function intact	332 (67.6%)
Class I	114 (23.2%)
Class II	39 (7.9%)
Class III	6 (1.2%)
Class IV	0 (0.0%)
Shock on EN initiation within 24 h of RICU admission	46 (12.2%)
Shock on EN initiation within 48 h of RICU admission	8 (1.8%)
Reasons for no EN feeding within 48 h	
Oral feeding	523 (90%)
Uncontrolled shock	21 (3.6%)
Uncontrolled upper gastrointestinal bleeding	21 (3.6%)
Threatening hypoxemia and acidosis	13 (2.2%)
Others	3 (0.5%)

AGI, Acute Gastrointestinal Injury.

### Type of EN route and EN formulation

Among 499 patients requiring EN support, nasogastric tube placement was utilized in 457 cases (91.6%), while a nasojejunal tube was inserted for 42 patients (8.4%). Continuous pump infusion constituted the predominant EN delivery method (82.1%), followed by intermittent pump-driven administration (7.6%) and gravity-controlled feeding protocols (4.4%).

Among the patients, 15.1% initially opted for elemental formulas, 68.3% received intact protein formulas, and 6.6% chose a homogenized diet. The prevalent choice among intact protein formulations was high-energy EN formulae (32.9%), followed by normal-energy formulae (26.9%) and low-energy formulae (11.8%). Only in 36 cases were changes in their EN formulation performed.

### EN complications

During EN administration, gastrointestinal complications occurred in 26.1% (130/499) of patients, with 5.0% (25/499) developing two distinct complications and 1.6% (8/499) manifesting three concurrent adverse events. The principal etiologies of enteral feeding intolerance comprised abdominal distension (33.1%), diarrhea (31.5%), and elevated gastric residual volumes (18.5%). EN discontinuation was required in 52.3% (68/130) of affected patients, with the predominant indications being persistent gastric residual volume elevation (35.3%) and refractory abdominal distension (20.6%; Table 3).

**Table 3:** EN intolerance and discontinuation during EN feeding.

Variables	Number (%)
EN intolerance (130)	
Abdominal distention	43 (33.1)
Abdominal pain	5 (3.8)
Diarrhea	41 (31.5)
Nausea	8 (6.2)
Vomiting	16 (12.3)
Reflux	18 (13.8)
Increased GRV(ml)	24 (18.5)
Aspiration	2 (1.5)
Abnormal bowel sound	5 (3.8)
Gastrointestinal hemorrhage	17(13.1)
Nasoenteric Tube Displacement	2 (1.5)
Nasogastric Tube	2 (1.5)
Constipation	1 (0.7)
EN discontinuation due to intolerance (68)	
Abdominal distention	14 (20.6)
Abdominal pain	3 (4.4)
Diarrhea	9 (13.2)
Nausea	5 (7.4)
Vomiting	11 (16.2)
Reflux	13 (19.1)
Increased GRV	24 (35.3)
Aspiration	2 (2.9)
Abnormal or absence of bowel sounds	6 (8.8)
Gastrointestinal hemorrhage	13 (19.1)
Nasotube intolerance	3 (4.4)
Feeding tube obstruction or translocation	3 (4.4)

GRV, gastric residual volume.

### Aspiration precautions

Among the cohort of patients initiating EN within the initial 7-day period, 79.8% (398/499) received feedings in the semi-recumbent position (head-of-bed elevation ≥30°), with the remaining cohort receiving feedings in supine positioning or suboptimal elevation angles. Gastrointestinal prokinetic agents were used in 28.5% of patients. Among 266 patients requiring invasive mechanical ventilation, 211 underwent endotracheal cuff pressure monitoring, with all measurements maintained within the clinically recommended range of 25–30 cmH2O. Standardized oral care protocols were administered to 98.4% of the cohort, with standard implementation twice daily throughout the intensive care stay (Table 4).

**Table 4:** Aspiration precautions for high aspiration risk patients.

Index	Results (%)
High aspiration risk	
Bed to care ratio <1:2.5	85 (17.1)
Mechanical ventilation or prolonged horizontal supine >72 h	270 (54.2)
Age >70 years old	263 (52.8)
Vomit	13 (2.6)
Past history of aspiration	64 (12.9)
Neuromuscular disease or structural abnormalities of the Aerodigestive tract	54 (10.8)
Decreased level of consciousness	219 (44.0)
Aspiration precautions	
Position	494 (99.2)
The head of the bed is raised ≥30°	398 (79.9)
The head of the bed is raised <30°	89 (17.9)
Horizontal Supine	7 (1.4)
Oral care Frequency (times/day)	490 (98.4) 2 (2,3)
Prokinetic agents	142 (28.5)
Mosapride	90 (63.4)
Metoclopramide	15 (10.6)
Itopride	15 (10.6)
Monitor of cuff pressure (≥25 cmH2O)	211 (100.0)

### Nutritional adequacy in RICU patients

**Figure 2:**
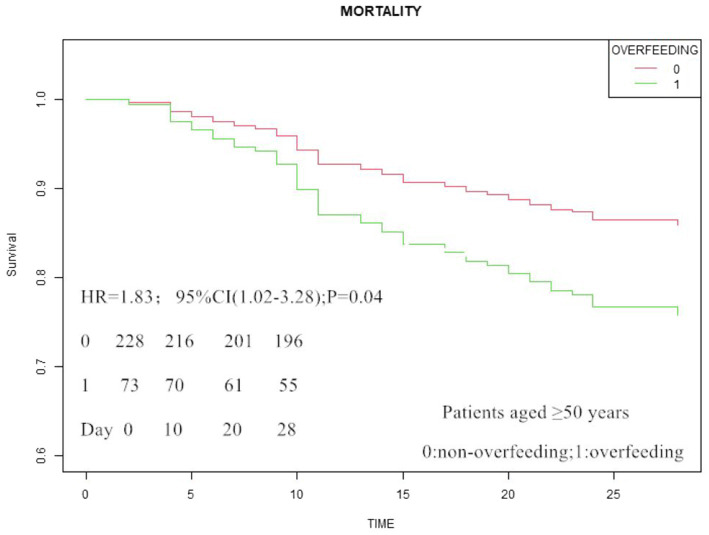
Cox analysis of overfeeding and 28-day mortality in patients with respiratory critical illness.

The mean nutritional adequacy for energy and protein provisions during post-RICU admission days 3–5 served as the primary metric for evaluating first-week nutritional attainment. In the analytical cohort of 317 patients with validated energy intake records, nutritional status stratification revealed: 103 underfed (32.5%), 137 adequately nourished (43.2%), and 77 overfed (24.3%) individuals. Multivariable logistic regression demonstrated significantly elevated non-social infection risks in overfed patients compared to both underfed counterparts (adjusted OR = 2.73, 95% CI 1.09–6.85; *p* = 0.03) and adequately feeding patients (adjusted OR = 2.65, 95% CI 1.16–6.06; *p* = 0.02; Table 5). No statistically significant difference was observed between patients with adequately feeding and underfeeding. However, no significant differences were detected among the three groups regarding 28-day mortality, duration of invasive ventilator use, or length of non-ICU hospital stay. Notably, in patients aged ≥50 years, after adjusting for age, APACHE II score, NRS2002 score, ALB and non-social infections, patients with overfeeding demonstrated a significantly increased risk of 28-day mortality compared to those with non-overfeeding (HR = 1.83, 95% CI [1.02–3.28], *p* = 0.04; Figure 2).

**Table 5:** The correlation between feeding adequacy and non-social infections in patients with respiratory critical illness.

	Model 1			Model 2		
	OR	95% CI	*p*	OR	95% CI	*p*
A. Comparison of the effects of overfeeding and underfeeding on non-social infections						
Underfeeding	1			1		
Overfeeding	2.48	1.12–5.50	0.02	2.73	1.09–6.85	0.03
B. Comparison of the effects of adequately feeding and overfeeding on non-social infections						
Adequately feeding	1			1		
Overfeeding	2.03	1.00–4.14	0.05	2.65	1.16–6.06	0.02

Model 1: Unadjust. Model 2: Adjust Gender; AGE; APACHEPFII; IPPVTIME; NRS2002.

Protein intake analysis in 298 evaluable patients revealed a cohort-wide mean provision of 0.9 g/kg/day. Suboptimal protein delivery (<1.2 g/kg/day) was observed in 218 cases (73.2%), while 80 patients (26.8%) met or exceeded the 1.2 g/kg/day threshold. Multivariable regression models identified no significant associations between protein adequacy levels and primary clinical endpoints (Supplementary Table 2; Supplementary Figure 1).

For the purpose of statistical analysis, patients were categorized into three groups based on their primary diagnosis: chronic obstructive pulmonary disease (COPD), severe pneumonia (including sepsis, septic shock, or ARDS), and others. The results indicated no significant intergroup differences in enteral nutrition intolerance rate, in-hospital infection rate, 28-day mortality, invasive mechanical ventilation, age, BMI, APACHE II, or NUTRIC scores (Supplementary Table 3).

## Discussion

This study, conducted in mainland China, represents the most extensive prospective observational study in RICUs. In 1,026 critically ill respiratory patients, this study assessed nutrition therapy delivery and the association of feeding adequacy with clinical outcomes during the first 7 days in the RICU.

Numerous studies highlight the benefits of early nutrition for high-risk patients (15–19). Doig et al.’s meta-analysis of six randomized controlled trials showed that early enteral nutrition (within 24 h of ICU admission) was associated with significant reductions in mortality (OR = 0.34, 95% CI 0.14–0.85) and pneumonia (OR = 0.31, 95% CI 0.12–0.78). Based on US costs, economic analyses revealed meaningful reductions in total care costs, with acute hospital care costs decreasing by US$14,462 per patient (95% CI US$5,464 to US$23,669). ASPEN and the Chinese expert consensus on nutrition advocated for early initiation of enteral nutrition within 24 or 48 h of ICU admission (6, 7). However, real-world epidemiological studies have shown less satisfactory results. A global study involving 9,777 patients from 46 countries reported that only 10% received enteral nutrition on the first day, increasing to over 40% after 5 days. The most extensive epidemiological study in Chinese general ICUs revealed suboptimal delivery of enteral nutrition, with proportions of patients starting enteral nutrition within 24, 48, and 72 h of ICU admission at 24.8, 32.7, and 40.0%, respectively (20).

Within our cohort of 1,026 RICU patients, EN was initiated in 378 cases (36.8%) within the initial 24-h period post-admission, increasing to 445 patients (43.4%) by 48 h, with cumulative EN implementation reaching 499 patients (48.6%) within the first week. Excluding patients who could take oral food, the proportion of patients who started EN within 24 and 48 h increased to 86.9 and 95.3%. Notably, complete nutritional deprivation persisted in only 14.8% (152/1,026) and 11.5% (118/1,026) of patients during the first 24-h and 48-h post-admission periods, respectively. The high percentage of patients in good nutritional condition within 48 h can be attributed to several factors. Patients admitted to RICUs were not severely ill, with only a quarter requiring invasive mechanical ventilation and over a third being able to eat orally. Additionally, the recognition and understanding of the importance of nutrition among RICU doctors likely contributed, partly due to the publication and promotion of the Chinese expert consensus on nutrition for critically ill respiratory patients (6).

In our study, the incidence of shock was 12.2% in patients who initiated EN within 24 h. Despite this, EN tolerance is acceptable. 26.1% of patients receiving EN developed complications, with 12.4% requiring discontinuation due to intolerance (e.g., high gastric residuals and abdominal distension). These patterns mirror findings from Chinese ICU cohort studies (20).

Previous studies have shown that both overfeeding and underfeeding negatively impact critically ill patients, particularly those at high nutritional risk (21–25). Inadequate calorie and protein intake have been reported in previous observational studies, with variations observed globally (26–28). For instance, a Canadian ICU survey found that 16% of patients received no nutrition support, and those who did received only 56 to 62% of estimated calorie and protein needs during the first 12 days (28). Similarly, a cross-sectional study in 116 Chinese ICUs showed that a minority of patients received over 80% of the estimated energy target within 24, 48, 72 h, and 7 days after ICU admission (20).

Within the analyzed cohort of patients with complete energy intake documentation (*n* = 317), nutritional delivery patterns during the initial 7-day post-RICU admission period comprised three distinct categories: suboptimal (32.5%, *n* = 103), adequate (43.2%, *n* = 137), and excessive (24.3%, *n* = 77). Multivariable analysis identified energy-overfed status as demonstrating significant associations with both non-social infection susceptibility and elevated mortality risk in geriatric subgroups. This pathophysiological mechanism appears mediated through nutrient restriction-induced autophagy upregulation, an evolutionarily conserved cytoprotective process that augments innate antimicrobial defenses. Conversely, excessive caloric provision may attenuate these protective pathways, potentially explaining the observed mortality risk elevation in overfed cohorts (29, 30). Stress-induced hyperglycemia represents a pathophysiologically conserved adaptive metabolic mechanism in critical illness, evolutionarily optimized to prioritize fuel substrate mobilization during systemic homeostatic challenges (31, 32). Overfeeding potentiates hyperglycemia and insulin resistance via impaired glucose homeostasis, consequently increasing susceptibility to non-social infections. This pathophysiological correlation was substantiated in the *EPANIC* trial, with mechanistic analysis suggesting caloric excess as a contributing determinant to infectious complications (33).

Whereas seminal works by Zusman et al. and Weijs et al. established both underfeeding and overfeeding as independent mortality predictors in ICU patients (34), our analysis revealed no significant mortality association with underfeeding status. This discordance likely reflects fundamental divergences in pathophysiological characteristics and illness severity between RICU and general ICU patient cohorts.

No statistically significant differences in clinical outcomes were observed between adequately fed and underfed patients, which is consistent with previous studies (35, 36). Notably, two large-scale randomized controlled trials (RCTs), the TARGET and PERMIT studies, demonstrated no significant differences in clinical outcomes among patients receiving low, normal, or high caloric intake (37, 38). Contrastingly, emerging evidence suggested reduced mortality risk in adequately feeding subgroups relative to underfed cohorts, potentially mediated by baseline nutritional risk stratification discrepancies (5, 39).

Critically ill patients typically require enhanced protein provision, with clinical studies indicating that protein intake exceeding 1.3 g/kg/day correlates with reduced 28-day mortality in mechanically ventilated patients. In contrast, standard protein supplementation at 0.8 g/kg/day shows no comparable survival benefit (40). In our cohort of patients with validated protein intake documentation, the mean protein provision during post-RICU admission days 3–5 was 0.9 g/kg/day. Crucially, our analysis demonstrated no significant mortality reduction with protein delivery exceeding either 1.2 g/kg/day (*p* = 0.31) or 1.3 g/kg/day (*p* = 0.42), replicating outcomes from the *EFFORT-Protein* trial (41).

Our study’s strengths include its prospective design, large sample size across 68 RICUs in China, and comprehensive parameter consideration. The detailed description of EN delivery within the first 7 days after ICU admission provides profound insights into the current EN status in Chinese RICUs. RICU patients often present with respiratory failure, pulmonary infections, sepsis, or septic shock, which significantly elevate their metabolic rate and place them in a hypercatabolic state. This leads to a sharp increase in energy and protein consumption. While inadequate nutritional support can result in rapid muscle wasting, overfeeding also poses significant risks. For patients with respiratory failure, diaphragmatic function is critical, especially in those aiming for extubation. Insufficient energy and protein intake can lead to weaning failure and impede recovery from respiratory failure. Conversely, excessive carbohydrate intake generates substantial CO₂ production. In patients with compromised ventilatory function, this increased CO₂ load can significantly elevate respiratory effort, potentially causing respiratory muscle fatigue, difficulty in weaning, or hypercapnia. This is particularly critical in patients with chronic obstructive pulmonary disease (COPD). Therefore, it is essential to develop individualized nutritional support strategies for RICU patients. These findings can inform improvement strategies across various RICUs, addressing existing issues in EN feeding.

However, there are several limitations to acknowledge. Firstly, this study was cross-sectional, which prevents establishing causal relationships. Secondly, as the study was conducted during the summer season, it may not reflect the typical patient types and severity in RICUs. Consequently, the study results might underestimate the number of high nutritional risk patients, the severity of patients, and the proportion of EN patients, showing a relatively higher proportion of oral feeding patients. Thirdly, the study’s methodological constraints stem from the limited cohort size of enterally-fed patients, restricting statistical power for analyzing EN initiation timelines, caloric/protein delivery accuracy, goal attainment, and feeding tolerance. Consequently, potential associations between underfeeding regimens and clinical prognosis remain undetermined, necessitating prospective investigations with expanded sample sizes to validate these preliminary findings. Additionally, in this investigation, caloric and protein requirements were calculated using established body weight-based equations to examine correlations between nutritional adequacy and clinical outcomes, without employing indirect calorimetry for target validation. While weight-based modeling offers practical advantages in clinical settings, subsequent research should prioritize validating these associations through indirect calorimetry-guided nutritional protocols. Lastly, it is essential to note that the findings of this study may not apply to other types of ICUs, as it was specifically conducted in RICUs.

In conclusion, early nutritional intervention initiation has become standard practice in Chinese RICUs, and the proportion of EN initiated within 48 h of RICU admission was optimistic. Our multicenter analysis substantiates that energy-overfed status were associated with elevated non-social infection incidence in critically ill respiratory patients and increased mortality risk only in subgroup over 50 years. These findings underscore the critical need for precision nutritional protocols incorporating individualized caloric requirement quantification and real-time delivery surveillance systems to balance therapeutic efficacy with iatrogenic overfeeding mitigation.

## Data Availability

The raw data supporting the conclusions of this article will be made available by the authors, without undue reservation.

## Supplementary material

SUPPLEMENTARY FIGURE 1 Association between protein intake and 28-day mortality.

## References

[1] Alberda C Gramlich L Jones N Jeejeebhoy K Day AG Dhaliwal R . The relationship between nutritional intake and clinical outcomes in critically ill patients: results of an international multicenter observational study. Intensive Care Med. (2009) 35:1728–37. doi: 10.1007/s00134-009-1567-4, 19572118

[2] De Jonghe B Bastuji-Garin S Durand MC. Respiratory weakness is associated with limb weakness and delayed weaning in critical illness. Crit Care Med. (2007) 35:2007–15. doi: 10.1097/01.ccm.0000281450.01881.d8, 17855814

[3] Lin YR Chen PC Li WT Huang MH Huang SF Wang CJ . The relationship between caloric intake and clinical outcomes in critically ill patients: a retrospective study. Clin Nutr ESPEN. (2025) 65:9–15. doi: 10.1016/j.clnesp.2024.11.008, 39551353

[4] McClave SA Taylor BE Martindale RG Warren MM Johnson DR Braunschweig C . Guidelines for the provision and assessment of nutrition support therapy in the adult critically ill patient: Society of Critical Care Medicine (SCCM) and American Society for Parenteral and Enteral Nutrition (a.S.P.E.N.). JPEN J Parenter Enteral Nutr. (2016) 40:159–211. doi: 10.1177/0148607115621863, 26773077

[5] Singer P Blaser AR Berger MM Alhazzani W Calder PC Casaer MP . ESPEN guideline on clinical nutrition in the intensive care unit. Clin Nutr. (2019) 38:48–79. doi: 10.1016/j.clnu.2018.08.037, 30348463

[6] Association PCoCCMRPSCMD, Association PaCCMgCSoTSCM. Expert consensus on nutritional support therapy for respiratory critically ill patients in China. Chin Med J. (2020) 100:573–85. doi: 10.3760/cma.j.cn112147-20220407-00290

[7] Compher C Bingham AL McCall M Patel J Rice TW Braunschweig C . Guidelines for the provision of nutrition support therapy in the adult critically ill patient: the American Society for Parenteral and Enteral Nutrition. JPEN J Parenter Enteral Nutr. (2022) 46:12–41. doi: 10.1002/jpen.2267, 34784064

[8] Kreymann KG Berger MM Deutz NE Deutz NEP Hiesmayr M Jolliet P . ESPEN guidelines on enteral nutrition: intensive care. Clin Nutr. (2006) 25:210–23. doi: 10.1016/j.clnu.2006.01.021, 16697087

[9] Singer P Blaser AR Berger MM Calder PC Casaer M Hiesmayr M . ESPEN practical and partially revised guideline: clinical nutrition in the intensive care unit. Clin Nutr. (2023) 42:1671–89. doi: 10.1016/j.clnu.2023.07.011, 37517372

[10] Heyland DK Dhaliwal R Day A Jain M Drover J. Validation of the Canadian clinical practice guidelines for nutrition support in mechanically ventilated, critically ill adult patients: results of a prospective observational study. Crit Care Med. (2004) 32:2260–6. doi: 10.1097/01.ccm.0000145581.54571.32, 15640639

[11] O'Leary-Kelley CM Puntillo KA Barr J. Nutritional adequacy in patients receiving mechanical ventilation who are fed enterally. Am J Crit Care. (2005) 14:222–31. doi: 10.4037/ajcc2005.14.3.22 15840896

[12] Peev MP Yeh DD Quraishi SA Osler P Chang Y Gillis E . Causes and consequences of interrupted enteral nutrition: a prospective observational study in critically ill surgical patients. JPEN J Parenter Enteral Nutr. (2015) 39:21–7. doi: 10.1177/0148607114526887, 24714361 PMC4402286

[13] Heyland DK Ortiz A Stoppe C Patel JJ Yeh DD Dukes G . Incidence, risk factors, and clinical consequence of enteral feeding intolerance in the mechanically ventilated critically ill: an analysis of a multicenter, multiyear database. Crit Care Med. (2021) 49:49–59. doi: 10.1097/ccm.0000000000004712, 33148950

[14] Zheng H Cai L Wang P Zheng L Lin J Sun T . Causes and impacts of interrupted enteral nutrition in critically ill patients: a secondary analysis of a cluster-randomized controlled trial. Nurs Crit Care. (2025) 30:e70006. doi: 10.1111/nicc.70006, 40069998

[15] Doig GS Heighes PT Simpson F Sweetman EA Davies AR. Early enteral nutrition, provided within 24 h of injury or intensive care unit admission, significantly reduces mortality in critically ill patients: a meta-analysis of randomised controlled trials. Intensive Care Med. (2009) 35:2018–27. doi: 10.1007/s00134-009-1664-4, 19777207

[16] Doig GS Chevrou-Séverac H Simpson F. Early enteral nutrition in critical illness: a full economic analysis using US costs. Clinicoecon Outcomes Res. (2013) 5:429–36. doi: 10.2147/ceor.S50722, 24003308 PMC3755543

[17] Artinian V Krayem H DiGiovine B. Effects of early enteral feeding on the outcome of critically ill mechanically ventilated medical patients. Chest. (2006) 129:960–7. doi: 10.1378/chest.129.4.960, 16608945

[18] Lee ZY Barakatun-Nisak MY Noor Airini I Heyland DK. Enhanced protein-energy provision via the enteral route in critically ill patients (PEP uP protocol): a review of evidence. Nutr Clin Pract. (2016) 31:68–79. doi: 10.1177/0884533615601638 26385874

[19] Hadi V Amiri Khosroshahi R Imani H Jahangirfard B Majari K Kiany F . Impact of early versus delayed enteral nutrition on ICU outcomes: a comparative study on mortality, ventilator dependence, and length of stay. Eur J Med Res. (2025) 30:315. doi: 10.1186/s40001-025-02579-3, 40259420 PMC12013059

[20] Xing J Zhang Z Ke L Zhou J Qin B Liang H . Enteral nutrition feeding in Chinese intensive care units: a cross-sectional study involving 116 hospitals. Crit Care. (2018) 22:229. doi: 10.1186/s13054-018-2159-x, 30244686 PMC6151932

[21] Wang CY Fu PK Huang CT Chen CH Lee BJ Huang YC. Targeted energy intake is the important determinant of clinical outcomes in medical critically ill patients with high nutrition risk. Nutrients. (2018) 10:1731. doi: 10.3390/nu10111731, 30423896 PMC6266394

[22] Compher C Chittams J Sammarco T Nicolo M Heyland DK. Greater protein and energy intake may be associated with improved mortality in higher risk critically ill patients: a multicenter, multinational observational study. Crit Care Med. (2017) 45:156–63. doi: 10.1097/ccm.0000000000002083, 28098623

[23] Patkova A Joskova V Havel E Kovarik M Kucharova M Zadak Z . Energy, protein, carbohydrate, and lipid intakes and their effects on morbidity and mortality in critically ill adult patients: a systematic review. Adv Nutr. (2017) 8:624–34. doi: 10.3945/an.117.015172, 28710148 PMC5502871

[24] Wang CY Huang CT Chen CH Chen MF Ching SL Huang YC. Optimal energy delivery, rather than the implementation of a feeding protocol, may benefit clinical outcomes in critically ill patients. Nutrients. (2017) 9:527. doi: 10.3390/nu9050527, 28531142 PMC5452257

[25] Wang L Long Y Zhang Z Lin J Zhou J Li G . Association of energy delivery with short-term survival in mechanically ventilated critically ill adult patients: a secondary analysis of the NEED trial. Eur J Clin Nutr. (2024) 78:257–63. doi: 10.1038/s41430-023-01369-6, 38007601

[26] Javid Z Shadnoush M Khadem-Rezaiyan M Mohammad Zadeh Honarvar N Sedaghat A Hashemian SM . Nutritional adequacy in critically ill patients: result of PNSI study. Clin Nutr. (2021) 40:511–7. doi: 10.1016/j.clnu.2020.05.047, 32711949

[27] Bendavid I Singer P Theilla M Themessl-Huber M Sulz I Mouhieddine M . NutritionDay ICU: a 7 year worldwide prevalence study of nutrition practice in intensive care. Clin Nutr. (2017) 36:1122–9. doi: 10.1016/j.clnu.2016.07.012, 27637833

[28] Heyland DK Schroter-Noppe D Drover JW Jain M Keefe L Dhaliwal R . Nutrition support in the critical care setting: current practice in Canadian ICUs--opportunities for improvement? JPEN J Parenter Enteral Nutr. (2003) 27:74–83. doi: 10.1177/014860710302700174, 12549603

[29] Tappy L Schwarz JM Schneiter P Cayeux C Revelly J-P Fagerquist CK . Effects of isoenergetic glucose-based or lipid-based parenteral nutrition on glucose metabolism, de novo lipogenesis, and respiratory gas exchanges in critically ill patients. Crit Care Med. (1998) 26:860–7. 9590315 10.1097/00003246-199805000-00018

[30] Asarian L Langhans W. A new look on brain mechanisms of acute illness anorexia. Physiol Behav. (2010) 100:464–71. doi: 10.1016/j.physbeh.2010.04.009, 20394763

[31] Marik PE Bellomo R. Stress hyperglycemia: an essential survival response! Crit Care. (2013) 17:305. doi: 10.1186/cc12514, 23470218 PMC3672537

[32] Chen L Zeng X Zou W Chen M Fan Y Huang P. Predictive performance of stress hyperglycemia ratio for poor prognosis in critically ill patients: a systematic review and dose-response meta-analysis. Eur J Med Res. (2025) 30:613. doi: 10.1186/s40001-025-02868-x, 40646634 PMC12247285

[33] Casaer MP Mesotten D Hermans G Wouters PJ Schetz M Meyfroidt G . Early versus late parenteral nutrition in critically ill adults. N Engl J Med. (2011) 365:506–17. doi: 10.1056/NEJMoa1102662, 21714640

[34] Zusman O Theilla M Cohen J Kagan I Bendavid I Singer P. Resting energy expenditure, calorie and protein consumption in critically ill patients: a retrospective cohort study. Crit Care. (2016) 20:367. doi: 10.1186/s13054-016-1538-4, 27832823 PMC5105237

[35] Arabi YM Aldawood AS Al-Dorzi HM Tamim HM Haddad SH Jones G . Permissive underfeeding or standard enteral feeding in high- and low-nutritional-risk critically ill adults. Post hoc analysis of the PermiT trial. Am J Respir Crit Care Med. (2017) 195:652–62. doi: 10.1164/rccm.201605-1012OC, 27589411

[36] Marik PE Hooper MH. Normocaloric versus hypocaloric feeding on the outcomes of ICU patients: a systematic review and meta-analysis. Intensive Care Med. (2016) 42:316–23. doi: 10.1007/s00134-015-4131-4 26556615

[37] Arabi YM Aldawood AS Haddad SH Al-Dorzi HM Tamim HM Jones G . Permissive underfeeding or standard enteral feeding in critically ill adults. N Engl J Med. (2015) 372:2398–408. doi: 10.1056/NEJMoa1502826, 25992505

[38] Chapman M Peake SL Bellomo R. Energy-dense versus routine enteral nutrition in the critically ill. N Engl J Med. (2018) 379:1823–34. doi: 10.1056/NEJMoa1811687 30346225

[39] Heyland DK Stephens KE Day AG McClave S. The success of enteral nutrition and ICU-acquired infections: a multicenter observational study. Clin Nutr. (2011) 30:148–55. doi: 10.1016/j.clnu.2010.09.011, 20971534

[40] Weijs PJ Sauerwein HP Kondrup J. Protein recommendations in the ICU: g protein/kg body weight - which body weight for underweight and obese patients? Clin Nutr. (2012) 31:774–5. doi: 10.1016/j.clnu.2012.04.007, 22640477

[41] Heyland DK Patel J Compher C Rice TW Bear DE Lee Z-Y . The effect of higher protein dosing in critically ill patients with high nutritional risk (EFFORT protein): an international, multicentre, pragmatic, registry-based randomised trial. Lancet. (2023) 401:568–76. doi: 10.1016/s0140-6736(22)02469-2, 36708732

