# Supplementary figures and images for: Nutritional practices and impact of feeding adequacy on clinical outcomes in Chinese respiratory intensive care units patients: a prospective observational study (ORIENT study)
Source: Front Nutr. 2026 Jan 20;12:1719386. doi: 10.3389/fnut.2025.1719386 (PMC12866611; doi:10.3389/fnut.2025.1719386)

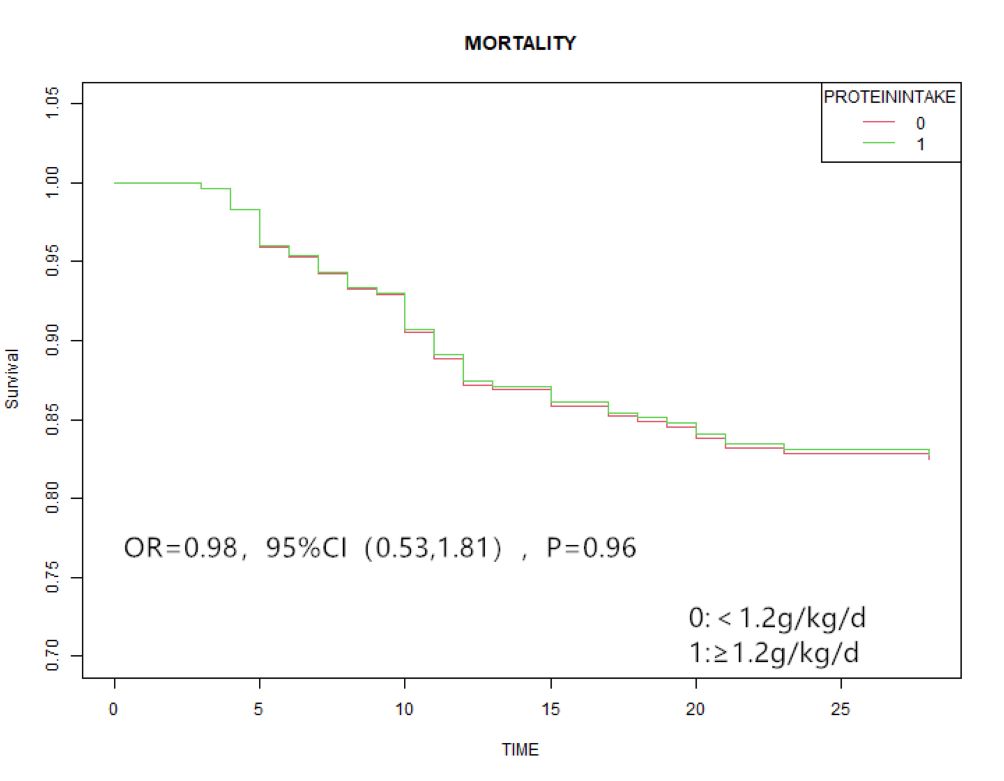

Supplement: SUPPLEMENTARY FIGURE 1 — Association between protein intake and 28-day mortality. [file Image_1.JPEG]
