# Supplementary material for: Nutritional practices and impact of feeding adequacy on clinical outcomes in Chinese respiratory intensive care units patients: a prospective observational study (ORIENT study)
Source: Front Nutr. 2026 Jan 20;12:1719386. doi: 10.3389/fnut.2025.1719386 (PMC12866611; doi:10.3389/fnut.2025.1719386)
Supplement: Supplementary file 2 [file Table_1.DOCX]

# eTable 1 Statistical Analysis Results of baseline ICU characteristics

| Index | Analysis Result |
| --- | --- |
| Hospital beds  Number  Median  Q1;Q3 | 68  2500  1753 ;3000 |
| ICU type  Number  RICU | 68  68(100.0%) |
| ICU beds /nurse ratio  Number  Mean ± SD | 68  0.63 ±0.19 |
| Professional dietitian  Number  Yes | 68  9( 13.2%) |
| Nutritional Care team  Number  Yes | 68  22( 32.4%) |

Notes: Q1: Quantile 25%; Q3: Quantile 75%
