# Supplementary material for: Nutritional practices and impact of feeding adequacy on clinical outcomes in Chinese respiratory intensive care units patients: a prospective observational study (ORIENT study)
Source: Front Nutr. 2026 Jan 20;12:1719386. doi: 10.3389/fnut.2025.1719386 (PMC12866611; doi:10.3389/fnut.2025.1719386)
Supplement: Supplementary file 3 [file Table_2.DOCX]

eTable2. Correlation analysis of protein intake and clinical outcomes in patients with respiratory critical illness

|  | OR | 95%CI | P |
| --- | --- | --- | --- |
| Nosocomial infection | 1.29 | (0.66,2.53) | 0.45 |
| Duration of invasive ventilator use | -0.76 | (-2.94,1.42) | 0.50 |
| Length of non-ICU hospital stay | -0.04 | (-2.40,2.33) | 0.97 |
