# Supplementary material for: Nutritional practices and impact of feeding adequacy on clinical outcomes in Chinese respiratory intensive care units patients: a prospective observational study (ORIENT study)
Source: Front Nutr. 2026 Jan 20;12:1719386. doi: 10.3389/fnut.2025.1719386 (PMC12866611; doi:10.3389/fnut.2025.1719386)
Supplement: Supplementary file 4 [file Table_3.DOCX]

|  | COPD(48) | Severs pneumonia(207) | Others(62) | P |
| --- | --- | --- | --- | --- |
| Age(years) | 75.08(12.55) | 73.46(12.88) | 70.55(13.79) | 0.161 |
| BMI(kg/m^2^) | 21.63(3.69) | 21.55(3.82) | 22.03(3.74) | 0.688 |
| APACHE II | 16.00(13,20) | 17.00(13,21) | 17.00(11,21) | 0.924 |
| mNutric | 4.00(3,5) | 4(3,5) | 4.00(3,5) | 0.904 |
| MV(Yes) | 24(50%) | 90(43.48%) | 23(37.10) | 0.396 |
| Mortality(Yes) | 8(16.67%) | 32(15.46%) | 14(22.58%) | 0.424 |
| Non-social(Yes) | 5(10.12%) | 36(17.39%) | 9(14.52%) | 0.468 |
| EN within 48h(Yes) | 46(95.83%) | 202(97.58%) | 58(93.55%) | 0.189 |

eTable3: Comparison of Basic Characteristics of Patients with Different Diseases

Data are expressed as mean(SD), or n (%).

BMI body weight index; APACHE acute physiology and chronic health evaluation; MV:mechanical ventilation;

EN:enteral nutrition
